# The adjacent positioning of co-regulated gene pairs is widely conserved across eukaryotes

**DOI:** 10.1186/1471-2164-13-546

**Published:** 2012-10-10

**Authors:** James T Arnone, Jeffrey R Arace, Sara Kass-Gergi, Michael A McAlear

**Affiliations:** 1 Department of Molecular Biology and Biochemistry, Wesleyan University, Middletown, CT 06459, USA; 2 Department of Computer Science, University of Colorado Boulder, Boulder, CO, 80309-0430, USA

## Abstract

**Background:**

Coordinated cell growth and development requires that cells regulate the expression of large sets of genes in an appropriate manner, and one of the most complex and metabolically demanding pathways that cells must manage is that of ribosome biogenesis. Ribosome biosynthesis depends upon the activity of hundreds of gene products, and it is subject to extensive regulation in response to changing cellular conditions. We previously described an unusual property of the genes that are involved in ribosome biogenesis in yeast; a significant fraction of the genes exist on the chromosomes as immediately adjacent gene pairs. The incidence of gene pairing can be as high as 24% in some species, and the gene pairs are found in all of the possible tandem, divergent, and convergent orientations.

**Results:**

We investigated co-regulated gene sets in *S. cerevisiae* beyond those related to ribosome biogenesis, and found that a number of these regulons, including those involved in DNA metabolism, heat shock, and the response to cellular stressors were also significantly enriched for adjacent gene pairs. We found that as a whole, adjacent gene pairs were more tightly co-regulated than unpaired genes, and that the specific gene pairing relationships that were most widely conserved across divergent fungal lineages were correlated with those genes that exhibited the highest levels of transcription. Finally, we investigated the gene positions of ribosome related genes across a widely divergent set of eukaryotes, and found a significant level of adjacent gene pairing well beyond yeast species.

**Conclusion:**

While it has long been understood that there are connections between genomic organization and transcriptional regulation, this study reveals that the strategy of organizing genes from related, co-regulated pathways into pairs of immediately adjacent genes is widespread, evolutionarily conserved, and functionally significant.

## Background

The ability of cells to appropriately regulate the expression levels of large sets of genes is one of the critical hallmarks of living systems, and it can be orchestrated across a wide range of circumstances, including during progression through the cell cycle, during cell differentiation and development, and in response to changing environmental conditions. For example, within a given cell cycle, cells regulate the biosynthesis of relevant sets of gene products that are appropriate for particular metabolic needs (i.e. the coordinated synthesis of histones during S phase [1]). Regulated expression can also extend over much longer time frames, as is the case for different members of the globin gene cluster, which are alternatively activated or repressed during mammalian development [2]. These regulatory changes can extend to hundreds of genes at a time, and can include subtle controls for maintaining a precise stoichiometry of gene product production. Cells can also rapidly respond to changing environmental conditions through large-scale transcriptional changes, as in the stress response in *S. cerevisiae*, which is associated with coordinated expression changes of roughly half of the genome [3].

One way that cells manage to coordinate the expression of large sets of genes is through the maintenance of particular sub-nuclear architectures. Indeed, perhaps the oldest and best characterized example of a sub-nuclear compartment is the nucleolus, the sub-nuclear localization where the rDNA is sequestered and production of the ribosome begins [4,5]. The rDNA repeats are transcribed in the nucleolus, and the nascent rRNAs are immediately subjected to extensive processing and assembly into pre-ribosomal particles [6]. Among the other sub-nuclear distinctions associated with eukaryotic genomes are the so called euchromatin and heterochromatin regions, which establish a local context that is either conducive or inhibitory to transcription, respectively [7]. More recently, it has been observed that there are dozens of sub-nuclear foci called ‘transcription factories’ that are enriched for actively expressed genes [8]. The localization of genes to particular sub-nuclear compartments can change quickly in response to environmental cues, where the activation of a gene can result in its re-localization to the nuclear periphery, allowing for coordination of transcription with processing and nuclear export [9].

There are localized subsets of the genome that are transcriptionally correlated in eukaryotic species as diverse as *A. thaliana*, *D. rerio*, *M. musculus, D. melanogaster,* and *S. cerevisiae*[10-14]. That is, physically adjacent DNA regions (typically a 2–3 gene window) tend to have a positive correlation of expression with each other. Additionally, the nematode worm *Caenorhabditis elegans* has operon-like structures, reminiscent of a prokaryotic genomic arrangement [15]. The distribution of genes throughout the genome is non-random, and the particular position of a gene on a chromosome can also play a critical role in its transcriptional regulation [16]. The globin and Hox genes are striking examples of this phenomenon, as their positional order in the genome corresponds to their spatial and temporal expression during development [2,17].

Multiple studies have found that the integration of a reporter construct in varied genomic locations can result in significant differences in its expression levels in many organisms, from yeast to humans [18-20]. More recently there has been an increased appreciation that this phenomenon is not limited to the insertion of an artificial reporter construct, but that local genomic context play an important role in gene regulation [12,21]. In particular, the effects of genomic position on transcription have been particularly well documented in *S. cerevisiae*, where the relocation of a gene from a euchromatic region to a heterochromatic region can result in repression of that gene [22]. The coordinated expression of adjacent genes is also important, particularly with those who share bi-directional promoters which allow for the coordinated production of two protein coding genes through a shared *cis-* regulatory region [23].

One of the most metabolically demanding pathways that growing cells must regulate is that of ribosome biogenesis, a complex biosynthetic pathway that depends on the coordinated action of the several hundred gene products required to produce functional ribosomes. Typically, the genes that function in ribosome biosynthesis are highly expressed, and they are also tightly regulated under changing environmental conditions. Previously, through our investigations in *S. cerevisae*, we described a large set of coregulated genes - the ribosome and rRNA biosynthesis (RRB) regulon – whose products function in various levels of rRNA and ribosome biosynthesis and processing. Like the genes whose products form the ribosomal proteins (RPs) themselves, the RRB genes are tightly co-regulated under changing cellular conditions [24,25]. Interestingly, we discovered that the genes from the RP and RRB gene sets exhibited an unusual pattern of their positions on the chromosomes; an unusually high fraction of the genes were found as immediately adjacent gene pairs [26]. We extended this observation across a wide variety of yeast species, including the finding that some 24% of the RRB genes from *C. albicans* are present as adjacent gene pairs, including all orientations of convergent, divergent and tandem gene arrangements [27].

In this study we report that high levels of paired adjacency for genes in regulated pathways is not limited to ribosome biogenesis in yeast. We observed that immediate gene adjacency is associated with tighter transcriptional co-regulation as compared to unpaired genes, and that as a whole, the set of paired genes are more tightly co-regulated. Elevated levels of gene adjacency can be observed across a diverse set of co-regulated gene sets in yeast, and many of the gene pairing relationships are conserved across divergent fungal lineages. Furthermore, we report that significant levels of immediate gene adjacency can also be found for ribosome biogenesis genes across a wide variety of eukaryotes. Together, these findings reveal a widespread and fundamental link in eukaryotes between adjacent gene placement and gene co-regulation.

## Results

### Gene pairing is associated with tighter transcriptional co-regulation within the RRB and RP regulons

In our previous analysis of the genes that are involved in ribosome biogenesis in *S. cerevisiae,* we noted that some 13% of the RP genes, and 15% of the RRB genes are located on the chromosomes as immediately adjacent gene pairs. Given recently updated gene annotations, we expanded the list of genes that comprise the RRB regulon, and included new members from the gene ontologies of ribosome biogenesis, rRNA processing, 90S pre-ribosome and the small subunit (SSU) processome. This expanded set brings the RRB family to 282 genes, of which 44 (16%) exist as immediately adjacent gene pairs.

**Figure 1:**
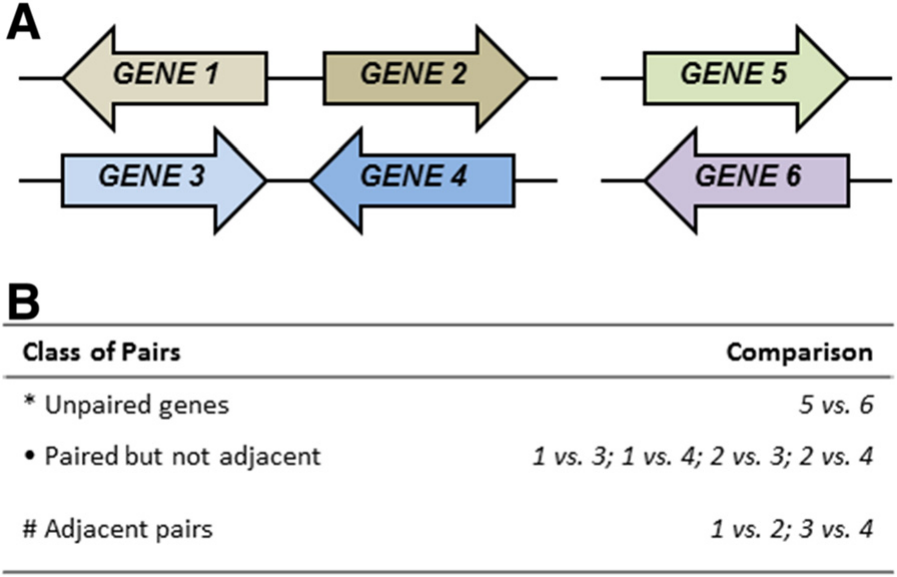
**Classification of pairing relationships compared in this study. **The members of a hypothetical regulon comprised of six genes (**A**) and the classification of the pairings used for the determination of the average Pearson’s correlation coefficient (**B**).

**Figure 2:**
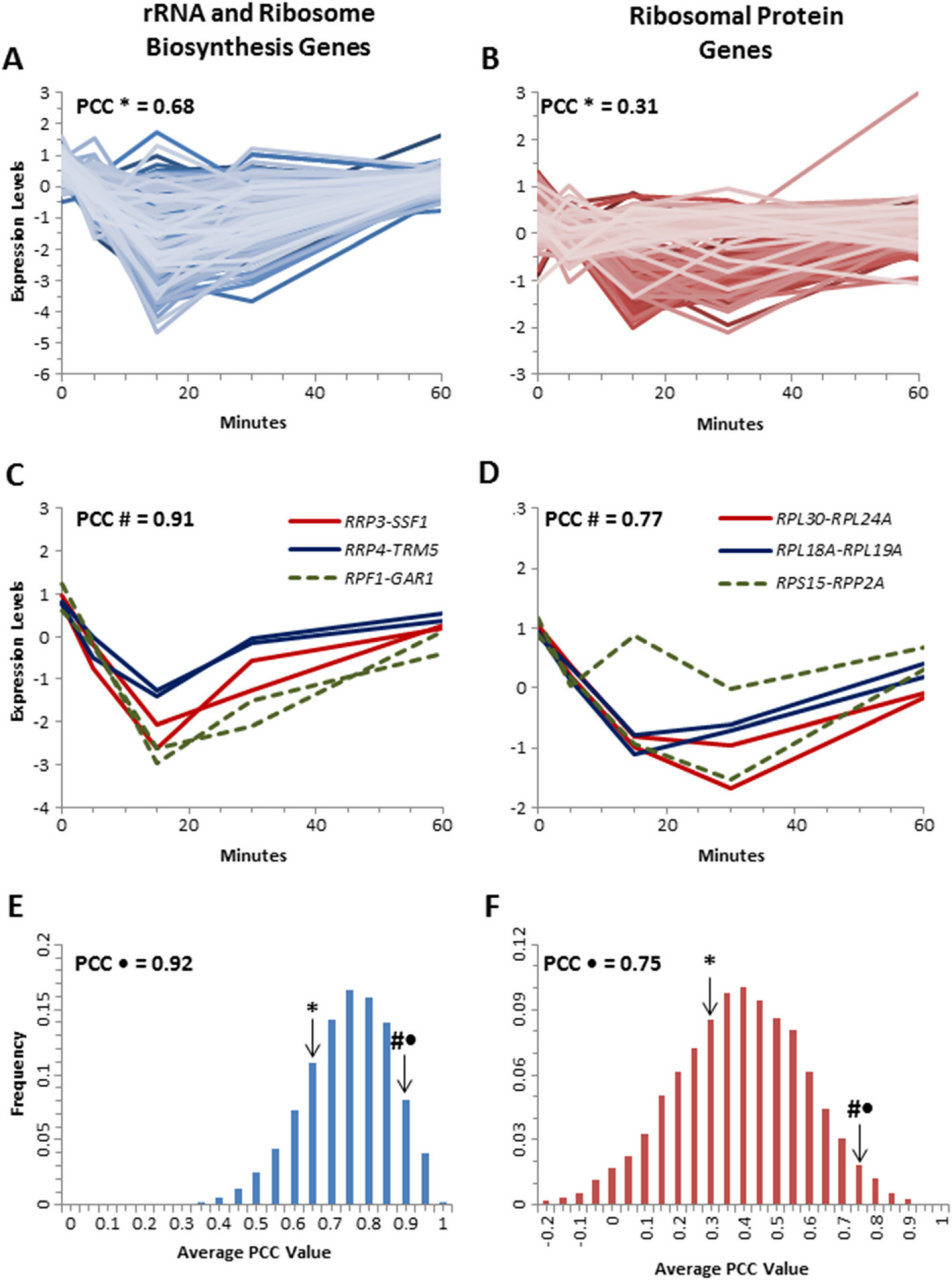
**Paired genes have a tighter transcriptional response throughout a heat shock time-course. **The transcriptional response of the unpaired RRB genes (n = 213) and RP genes (n = 151) throughout a heat-shock time-course (**A** and **B**). Representative expression profiles for three representative adjacent paired RRB genes and RP genes during this stressor (**C** and **D**). The average PCC was determined by bootstrapping random pairings and the frequency is plotted for the RRB genes (**E**) and the RP genes (**F**). The actual average PCC for the unpaired genes is indicated by *, for the paired but not adjacent genes by ·, and the adjacent paired genes by #.

To investigate what functional significance may be associated with this high degree of adjacent gene pairing, we investigated the transcriptional response of the RRB and RP genes following a 37°C heat-shock [3]. We had previously demonstrated that the members of the RRB and RP regulons exhibit a characteristic quick, and significant decline in expression levels following the stress of a heat shock. For this analysis, we investigated whether there were discernible differences in the transcriptional responses between the genes that were either paired, or present on their own across the yeast genome. For comparisons between gene expression profiles, we considered the members of the RRB or RP regulons that are unpaired (that is not adjacent to another gene in the regulon), the members from the set of paired but not adjacent genes, and comparisons between the two genes that form an adjacent pair (Figure 1). We analyzed the expression profiles, and calculated the Pearson’s correlation coefficient (PCC) for the various gene expression profile comparisons for the genes comprising the RRB and RP regulons (Figure 2). The correlation for every possible pairwise comparison in the set of 238 unpaired RRB genes was calculated, and the average was found to be 0.68, indicating that, overall, these genes exhibit similar expression responses (Figure 2A). In contrast, when we looked at the expression comparisons between the two genes that compromise an adjacent pair, the average PCC was 0.91, suggesting that adjacent genes tend to be very highly co-regulated (Figure 2C, and Additional file 1: Figure S1). Interestingly, the average PCC was similarly high (0.92), when we compared the expression profiles of RRB genes that were among the paired set, but not being compared to their immediately adjacent neighbor. To assess the statistical significance of these different PCC values, we used a bootstrapping approach to determine the average PCCs of random gene combinations of a size corresponding to the number of adjacent gene pairs from the RRB gene set, from out of the total number of RRB genes. The frequency histograms were determined from at least 10,000 iterations (Figure 2E). We noted a significantly elevated level in the PCC values for the adjacent and non-adjacent gene pairs as compared to the unpaired genes (P = 0.029). Similarly, for the RP regulon, the 156 unpaired RP genes exhibited and average PCC of 0.31, indicating that, as a group, they are less tightly regulated than the RRB genes (Figure 2B). The average PCC for the set of adjacent gene pairs was 0.77 (Figure 2D and Additional file 2: Figure S2), and that for the paired but not adjacent genes was 0.75. Again, a bootstrapping statistical analysis (Figure 2F) indicated that the adjacent and non-adjacent gene pairs exhibited significantly higher PCC values than would be expected by chance alone (P = 0.014).

**Figure 3:**
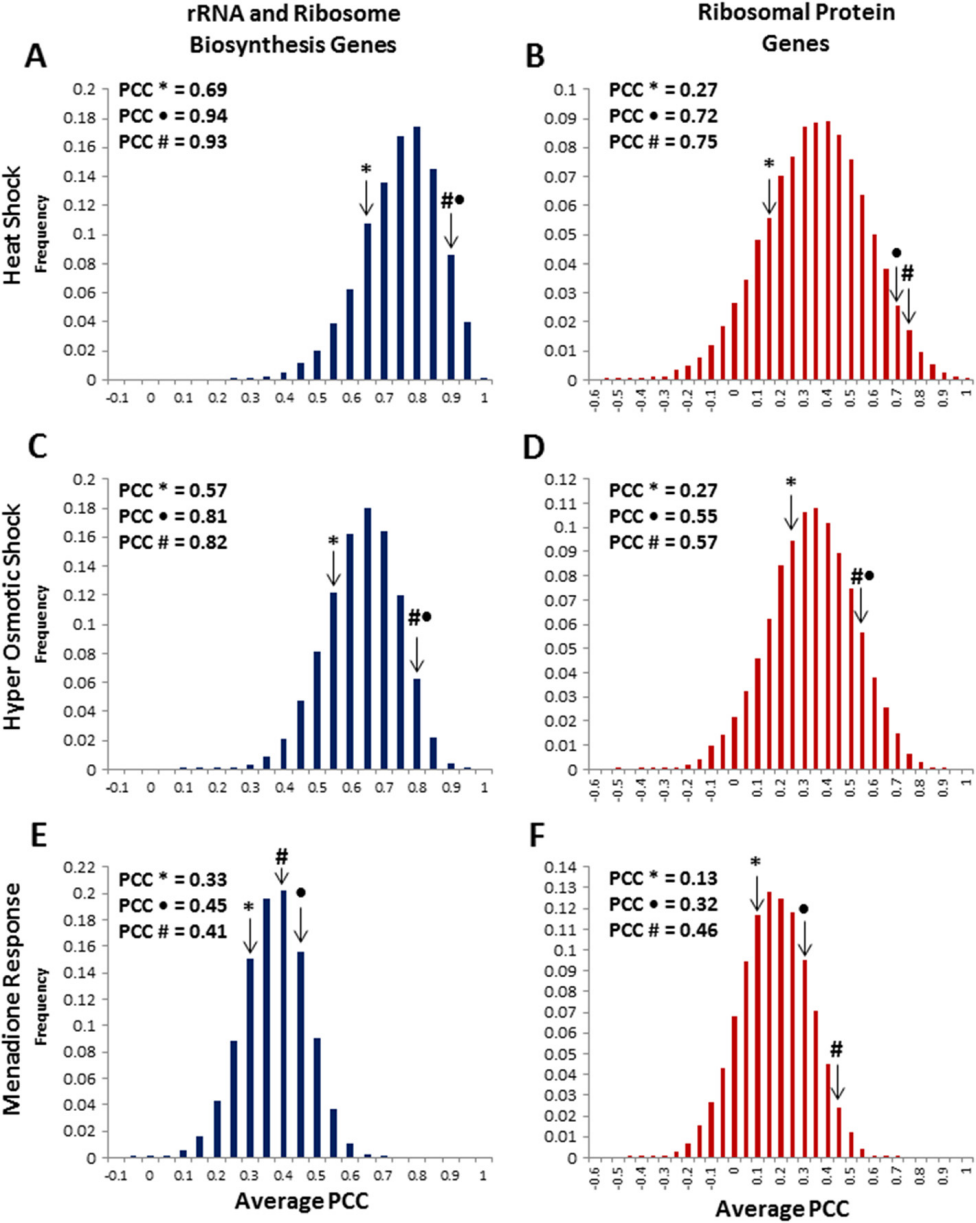
**Paired genes exhibit a tighter transcriptional coordination during the budding yeast stress response. **The average PCC was determined by bootstrapping random pairings of genes from the RRB and RP gene sets during a heat-shock (**A** and **B**), during the hyper-osmotic shock (**C** and **D**), and following treatment with menadione (**E** and **F**). The actual average PCC for the unpaired genes is indicated by *, for the paired but not adjacent genes by ·, and the adjacent paired genes by #.

In order to determine whether the gene pairs exhibit tighter, coregulated expression across different environmental conditions, we calculated the PCC values across an independent heat shock experiment, as well as in response to osmotic shock, and menadione exposure [3,28]. The PCC values were determined for the unpaired, the paired but not adjacent, and the adjacent RRB gene pairs across these conditions (Figure 3 A, C, and E), and we observed elevated levels of coregulation in the adjacent and non-adjacent gene pair sets. A similar bootstrapping analysis was used to determine the average PCC values for comparisons between two random genes within these sets, and the significance of the increased correlations (P = 0.010, 0.014 and 0.27 for heat shock, osmotic shock, and menadione treatment respectively). The RP genes were subjected to a similar analysis (Figure 3 B,D, and F), and again, we found significantly increased levels of coregulation in the set of adjacent and non-adjacent gene pairs as opposed to random genes (P = 0.019, 0.067, and 0.014 for heat shock, osmotic shock, and menadione treatment respectively).

**Figure 4:**
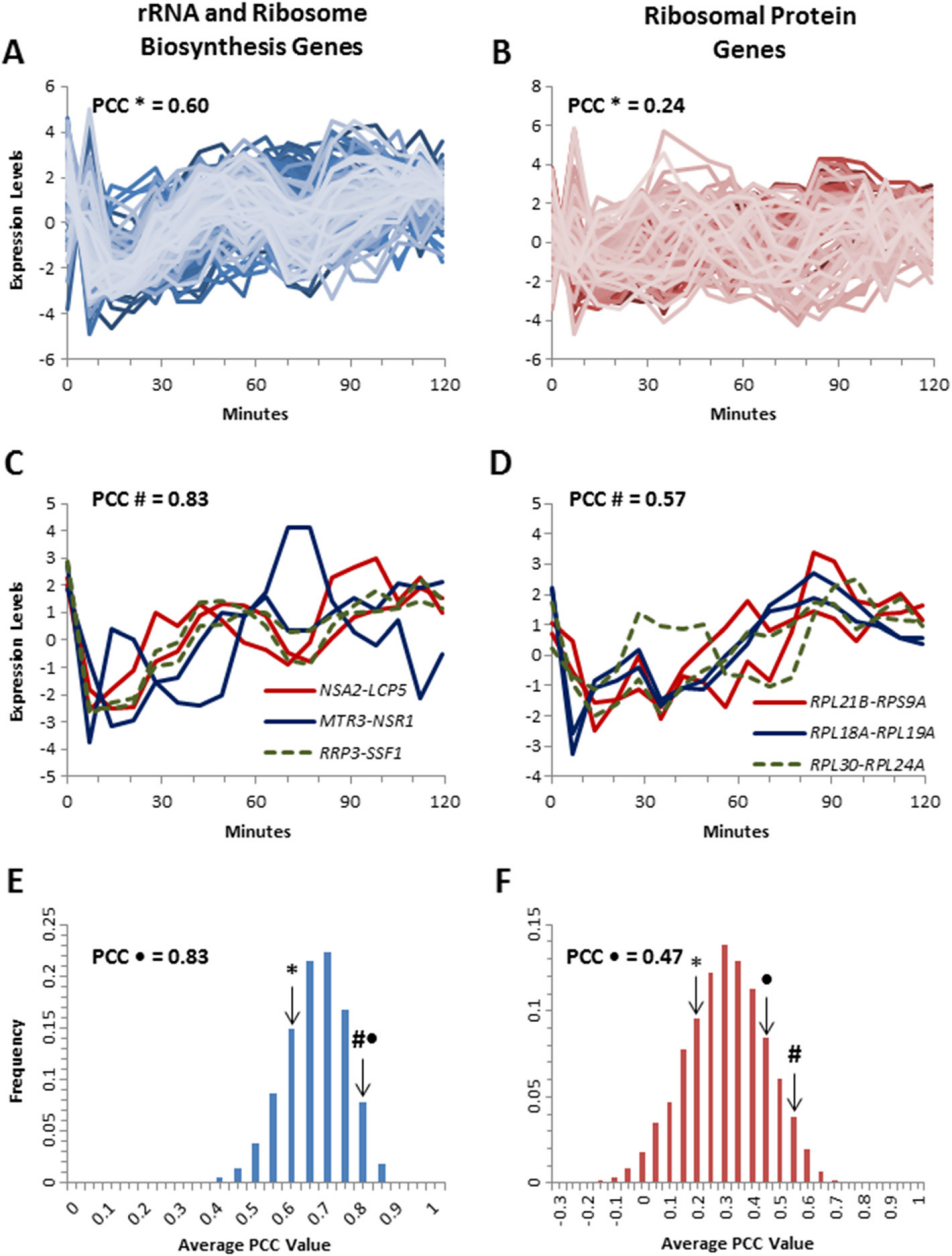
**Paired genes have a tighter transcriptional response following the release from alpha-factor arrest and progression through the cell-cycle. **The transcriptional response of the unpaired RRB genes (n = 210) and RP genes (n = 154) following release from alpha-factor (**A** and **B**). Representative expression profiles for three adjacent paired RRB and RP genes during this stressor (**C** and **D**). The average PCC was determined by bootstrapping random pairings of genes from the RRB (**E**) and the RP genes (**F**). The actual average PCC for the unpaired genes is indicated by *, for the paired but not adjacent genes by ·, and the adjacent paired genes by #.

To extend this analysis beyond conditions of gene repression, we analyzed expression profile comparisons associated with the induction of RRB and RP genes following release from alpha factor arrest [29]. The transcription profiles were plotted for the unpaired and paired members of the RRB and RP regulons, and the PCC values were calculated for every gene combination (Figure 4). The unpaired RRB genes had an average PCC of 0.60 (Figure 4A), the immediately adjacent pairs had an elevated PCC of 0.83 (P = 0.012) (Figure 4C and Additional file 3: Figure S3), and the set of paired but non-adjacent genes had an average PCC of 0.83 (Figure 4E). Again, the unpaired RP genes had a slightly lower average PCC of 0.24 (Figure 4 B), the immediate adjacent pairs have a PCC equal to 0.57 (P = 0.018) (Figure 4D and Additional file 4: Figure S4) and the paired but not adjacent set have a PCC of 0.47 (Figure 4 F). Thus, it appears that the paired set of RRB and RP genes have a significantly distinct expression response to changing cellular conditions, and while the immediately adjacent gene pairs tended to exhibit the highest levels of co-regulation, the whole set of genes that are paired appear to be much more tightly co-regulated as a group than the unpaired genes.

### Gene adjacency is conserved across different functional classes of genes in *S. cerevisiae*

Given that we found that a statistically significant fraction of the ribosome biogenesis genes are located on the genome as adjacent gene pairs, we sought to determine whether this non-random pattern of gene location was common to other sets of related genes. We initially incorporated the property of transcriptional co-regulation as one of the fundamental parameters that defined gene membership within the RRB regulon, and transcriptional co-regulation was previously known of the genes in the RP regulon. We sought to expand our analysis of the frequency of gene adjacency to include sets of related genes that were associated with a common transcriptional response. One such approach is to take advantage of the gene ontology groupings that have been developed concomitant with the increasing annotation of the complete set of genes from *S. cerevisiae* (as taken from the *Saccharomyces* Genome Database, accessed December 2011). The occurrence of gene adjacency from within different GO classes of genes was determined by mapping the genomic distribution of genes within these related biological processes. We chose gene sets that extended beyond ribosome biogenesis, and represented diverse cellular functional classes responding to intra- and intercellular stressors, as well as multiple biosynthetic processes and areas of metabolism (see Additional file 5 for the gene sets). The sets also ranged widely in gene number, ranging from the 8 member gene set of the purine biosynthetic pathway, to the 175 genes involved in the DNA damage response. We could observe a significant occurrence of immediately adjacent gene pairing in sets of genes that are involved in very different cellular processes, including diverse areas of metabolism and certain stress responses (Table 1). There is significant adjacent gene pairing (P < 0.05) observed amongst the genes that fall into the carbohydrate metabolism (10%), nitrogen compound metabolism (9%), purine base metabolism (62%), the DNA damage response (9%), and the heat shock response (22%) gene sets. As was observed for the RRB and RP gene sets, it was not the case that the adjacent genes were found exclusively in the divergent orientation, as tandem and convergent gene pairs were well represented. There were also many gene ontology classes associated with diverse areas of cellular metabolism that did not contain a significant fraction of adjacent gene pairs (Additional file 6: Table S1), including alcohol metabolism (GO #0006066), cellular respiration (GO #004533), metabolism of phosphorus (GO #0006793) and sulfur (GO #0006790), the response to acids (GO #0001101), osmotic stress (GO #0006970), oxidative stress (GO #0034599), and the unfolded protein response (GO #0006986).

**Table 1:** **Significant gene adjacency is conserved among several gene families in *S. cerevisiae***

**Ontology**	**G.O. Number**	**No. Genes**	**Adj. Genes**	**P-Value**	**Divergent**	**Tandem**	**Convergent**
Carbohydrate Metabolism	0005975	91	9	5.5x10^-4^	3	2	1
Nitrogen Metabolism	0006807	86	8	9.9x10^-4^	1	2	1
Purine Base Metabolism	0006144	8	5	1.2x10^-14^	1	2	0
DNA Damage Response	0006974	175	16	3.2x10^-2^	3	4	1
Response to Arsenic	0046685	8	3	4.8x10^-7^	1	1	0
Heat Shock Response	0009408	18	4	7.3x10^-8^	1	1	0
Response to Toxin	0009636	27	7	1.0x10^-10^	0	1	3

### Paired genes exhibit tighter transcriptional co-regulation in other regulons

To determine if the adjacent gene pairs from these additional regulons were also associated with tighter gene co-regulation, we compared their relative expression along with the unpaired genes under changing conditions. For example, we investigated the expression profiles of the 18 heat shock responsive genes following a heat shock time-course (Additional file 7: Figure S5). The paired heat shock genes show much higher average correlations to each other than did the unpaired genes during the heat-shock (PCC equal to 0.89 for paired genes compared to 0.14 for the unpaired genes). This pattern held true for a number of the other gene ontology groups, including a higher degree of paired gene co-regulation for those genes involved in carbohydrate metabolism (average PCC of 0.49 versus 0.17), purine base metabolism (average PCC of 0.13 versus −0.27) and nitrogen metabolism (a PCC equal to 0.14 versus 0). Therefore, it appears that the tighter co-regulation of paired genes can be observed across diverse gene sets.

### The high incidence of immediate gene adjacency is not the result of gene duplications

One mechanism whereby adjacent genes that function in the same biochemical pathway could arise is through a gene duplication event followed by subsequent divergence of one of the duplicates. Indeed, ancestral to many yeast lineages, including *S. cerevisiae,* there was a whole genome duplication event some 150 million years ago that was subsequently followed by the elimination of most of the duplicated genes [30]. This large scale doubling would first create duplicates on separate chromosomes, but potentially, genetic recombination, and subsequent elimination and modification of genes could give rise to high levels of adjacent genes that function in related pathways. In particular, because the majority of the RP genes from *S. cerevisiae* are present in the genome as two nearly identical homologs, we investigated the extent to which gene duplications could account for the high number of immediately adjacent genes that function in a given cellular pathway. To investigate this possibility, each member of the immediately adjacent gene pairs was compared by BLAST analysis to its adjacent partner and to the other genes in the *S. cerevisiae* genome [31]. Overwhelmingly, we found that the two members of an adjacent gene pair were not related to each other by sequence similarity, with the exception of one gene pair from the carbohydrate metabolism gene set. The tandem, adjacent gene pair *CDA1-CDA2* do appear to be derived from a gene duplication event, as they share extensive sequence similarity (E-value = 3.4x10^-94^ by BLAST). For every other comparison between the adjacent gene pairs, the E-value >1, and for those genes that did have a closely related homolog, it was located at another chromosomal location (see Additional file 8: Table S2). Thus, the high degree of adjacent gene pairing was not due to gene duplication events.

Interestingly, we did observe an adjacent pairing of RP genes that was related to the genome duplication event, but the adjacent pairing appears to have been present before the WGD, and has been conserved since. The adjacent *RPL18A-RPS19A* gene pair is found on chromosome 15 and the adjacent *RPL18B-RPS19B* gene pair is found on chromosome 14. While the *RPL18A* and *RPL18B* genes are highly related (E-value = 2.3x10^-81^) and the *RPS19A* and *RPS19B* genes are highly related (E-value = 2.6x10^-80^), in each case the immediately adjacent gene partners are not related (E-value>1).

### Adjacent gene pairings are conserved throughout widely divergent fungal lineages

**Figure 5:**
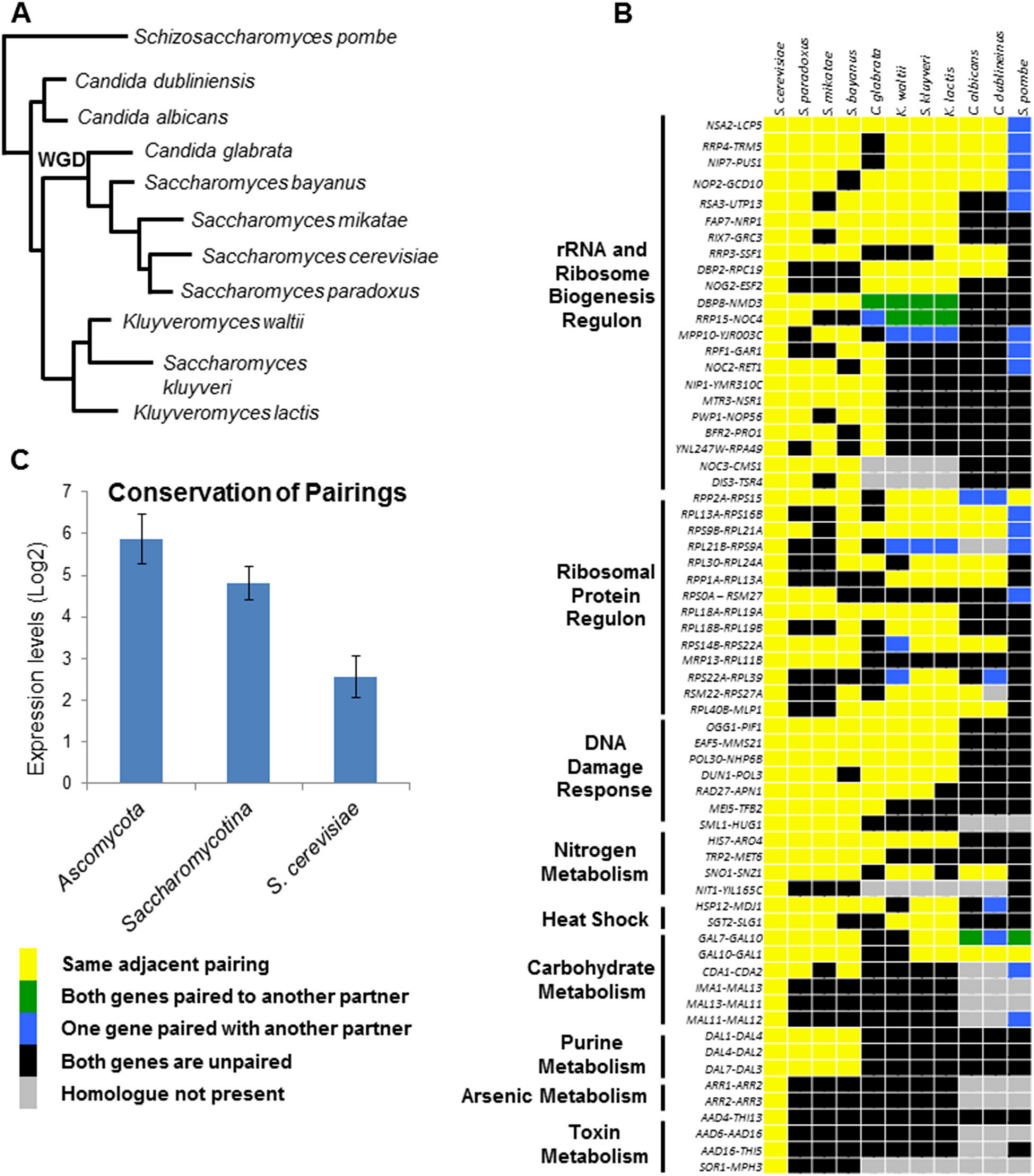
**The *S. cerevisiae *gene pairing relationships are conserved across divergent fungal lineages. **The phylogenetic relationship of fungal lineages analyzed in this study (**A**) and the heat map depicting the degree of conservation (**B**). Absolute mRNA levels of conserved gene pairing relationships (**C**).

To understand the evolutionary significance of the phenomena of adjacent gene pairing, we compared the conservation of these gene pairings across evolutionary divergent species within the phylum *Ascomycota*. These species include the closely related *Saccharomyces sensu strictu* species (*S. paradoxus, S. mikatae*, and *S. bayanus*), *C. glabrata*, the pre-WGD divergent species *K. lactis, K. waltii* and *S. kluyveri*, the human pathogenic yeasts *C. albicans* and *C. dubliniensis*, and the more distantly related fission yeast *S. pombe* (Figure 5A). We began this analysis by considering the adjacent gene pairs as they are found in *S. cerevisiae*, and followed the sets of genes to determine whether or not they were also paired – either to the same genes, or others - in the other yeast species.

For the RRB gene pairs, 20 out of the 22 gene pairs are also found as adjacent pairs in at least one of the *Saccharomyces sensu strictu* species, and 6 of the genes are paired within *C. albicans* and *C. dubliniensis*. For two of the gene pairs*, DBP8-NMD3* and *RRP15-NOC4*, both genes exist as partners with RRB genes in *K. lactis, K. waltii* and *S. kluyveri* although in each case the pairing is to a different partner. Although none of the same RRB gene pairs from *S. cerevisiae* are found in *S. pombe*, 8 of the paired RRB genes can be found paired with another RRB gene in *S. pombe*. For the RP regulon, 12 out of 14 RP gene pairings are the same in at least one *Saccharomyces sensu strictu* species, and 7 of the pairings are conserved in *C. albicans*. One gene pair, *RPP2A-RPS15*, is the same in *S. pombe*, and additionally there are four RP genes in *S. pombe* that are paired with a new RP gene.

All 8 of the gene pairs whose proteins function in the DNA damage response pathway are conserved in at least one *Saccharomyces sensu strictu* species, while only one pair is conserved through the *C. albicans* and *C. dubliniensis* lineages. None of these gene pairs are found in any sort of pairing arrangement in *S. pombe*. The gene parings that are involved in purine base metabolism are completely conserved only in the *Saccharomyces sensu strictu* species. Of the gene pairings that are observed among the carbohydrate metabolism, only the *GAL1*, *GAL7, GAL10* genes are found as immediate adjacent neighbors in species other than *S. cerevisiae*. In *S. pombe* the *GAL7-GAL10-GAL1* gene triplet contains an insertion of *SPBPB2B2.11* (a nucleotide sugar dehydrogenase involved in galactose metabolism) between *GAL7* and *GAL10*. The heat-shock response gene pairings, *HSP12-MDJ1* and *SGT2-SLG1*, are both conserved in *S. paradoxus, S. mikatae*, *K. lactis,* and *S. kluyveri.* The least conserved pairings are those from the ontology classes involved in the response to toxins and the response to arsenic, where none of the *S. cerevisiae* pairings are retained in any of the species investigated in this study, not even the closely related *S. paradoxus* (Figure 5B).

In order to assess the significance of the conservation of specific gene pairings across related yeast species, we investigated the background levels of small-scale gene pair synteny across four species. We used 10,000 iterations of a bootstrapping approach to query what fraction of either a random set of either 180 or 282 (the sizes of the RP and RRB regulons) *S. cerevisiae* genes were maintained as adjacent gene pairs in at least one of the *S. paradoxus*, *S. bayanus* and *S. mikatae* species*.* Overall, we found that there is a roughly 67% chance that a given adjacent gene pair from *S. cerevisiae* would be maintained as an adjacent gene pair in one of these three species ( Additional file 9: Figure S6). While this result demonstrates the overall high degree of synteny between the four yeasts, we observed that the adjacent gene pairs from the RP and RRB regulons were even more likely to be maintained as adjacent gene pairs within at least one of these three other species (85% and 91% maintenance for the RP and RRB adjacent gene pairs respectively). Therefore, there appears to be a selective pressure to maintain the adjacency of coregulated genes from ribosome-related metabolic pathways across divergent species.

We have previously reported that there are greater numbers of paired RRB and RP protein genes in both *C. albicans* and *S. pombe* than in *S. cerevisiae*[27]. We repeated this comparative analysis by using the *C. albicans* gene pairings as our starting reference set of genes and curated the conservation in both *C. dubliniensis* and *S. cerevisiae*. Again, we found broad conservation of gene pairing across many yeast species, there is absolute conservation of the RRB and the DNA damage gene pairings between *C. albicans* and *C. dubliniensis* and of the conserved RPs between these species, there is only one pairing that is not retained in C*. dubliniensis*.

To understand why certain gene pairs may be conserved to a higher degree across different yeast species than other gene pairings, we looked for a relationship between overall expression levels and high degrees of paired conservation. For our minimally conserved gene set, we grouped together the gene pairs that were only paired in *S. cerevisiae* (9 gene pairs). For our widely conserved gene set, we grouped together the genes in which at least one of the pairs was also paired all through the *Ascomycotina* (at least one of the genes is paired through to *S. pombe,* there were 17 pairs). The remaining gene pairs, which were conserved among *Saccharomycotina*, represent an intermediate level of conservation (38 gene pairs). We compared the overall expression levels of the three gene sets [32] and found that greater conservation correlates with higher levels of transcription, with the transcription levels of the most highly conserved genes being twice that of the genes that are not conserved (Figure 5C).

### The RRB and RP genes are found as immediate adjacent pairs across eukaryotes

To determine the extent to which adjacent gene pairing is conserved across more diverse eukaryotic species, we investigated the gene positions in other fungi (*N. crassa* and *A. nidulans*), in flagellates (*G. lamblia* and *N. gruberi*), in the fruit fly *D. melanogaster*, the nematode worm *C. elegans*, the flowering mustard plant *A. thaliana*, the ciliate *T. thermophila*, the malarial parasite *P. falciparum* as well as in humans (*H. sapiens*). Because the ribosomal protein genes are well characterized in these organisms, we could easily determine their genomic distribution and assess the incidence of adjacent gene pairing (Table 2, and Additional file 10).

**Table 2:** Immediate gene adjacency is conserved across widely divergent eukaryotes

**Species**	**Protein Coding Genes**	**Ribosomal Protein Genes**	**Adjacent RPs**	**P-Value**	**RRB Genes**	**Adjacent RRBs**	**P-value**
*S. cerevisiae*	5,797	180	24	1.1x10^-4^	282	44	4.1x10^-4^
*N. crassa*	10,082	115	22	3.9x10^-15^	*N/A*	*N/A*	*N/A*
*A. nidulans*	9,541	132	16	1.5x10^-7^	*N/A*	*N/A*	*N/A*
*H. sapiens*	22,287	144	6	2.8x10^-3^	118	0	7.1x10^-1^
*D. melanogaster*	13,601	165	6	1.1x10^-1^	110	0	8.3x10^-1^
*C. elegans*	19,735	86	6	1.1x10^-5^	106	6	1.6x10^-4^
*A. thaliana*	26,207	387	46	1.0x10^-14^	111	2	6.9 x10^-2^
*T. thermophila*	27,424	63	6	1.9x10^-8^	92	2	2.4x10^-2^
*P. falciparum*	5,268	160	21	2.4 x10^-4^	100	4	6.0x10^-4^
*G. lamblia*	6,470	66	2	1.5x10^-1^	94	0	3.2x10^-1^
*N. gruberi*	15,727	130	2	3.6x10^-1^	*N/A*	*N/A*	*N/A*

The number of ribosomal proteins varies significantly across eukaryotic species, ranging from the 66 genes that have been identified in *G. lamblia,* to 387 that can be found in *A. thaliana*. We found that the incidence of immediate gene adjacency for the ribosomal proteins varied widely across eukaryotes, and with immediately adjacent gene pairs representing fewer than 2% of the total in *N. gruberi* to over 13% in *P. falciparum*. The incidence of gene adjacency in the fungi *N. crassa* (19%) and *A. nidulans* (12%) is similar to that seen in *S. cerevisiae* (13%). There are also significant levels of RP gene adjacency seen in the widely studied model systems, *A. thaliana* (12%), *D. melanogaster* (4%), and *C. elegans* (7%).

Unlike the ribosomal proteins, the rRNA processing and ribosome biogenesis genes are less well characterized in each of the species that were studied. We set to first identify RRB genes in other species, and then to characterize their genomic organization including the conservation of adjacent gene pairing. The BLAST algorithm was used to identify homologues from 100 *S. cerevisiae* RRB genes in each species, and then we mapped their genomic distributions. We were able to identify between 92 (*T. thermophila*) and 118 (*H. sapiens*) RRB genes in each of the species that we analyzed. Due to the as yet incomplete genome assemblies for *N. crassa*, *A. nidulans*, and *N. gruberi* these species were omitted from this RRB analysis. While this approach would be expected to yield a vast underestimate of the degree of RRB gene pairing in other organisms since it is limited by a small sampling set (i.e. based on only 100 RRB genes from *S. cerevisiae*), and by the poor annotation records of RRB genes in general as compared to RP genes, we could, however, see evidence for adjacent gene pairing of RRB genes in other eukaryotes including 4% in *P. falciparum* and 6% in *C. elegans*. Interestingly, we did not observe significant levels of pairing between the members of the RRB and RP gene sets. Thus, it appears that significant levels of immediate, adjacent pairing of genes related to ribosome biogenesis are widely conserved across diverse eukaryotic lineages.

## Discussion

### Significant adjacent gene pairing is found across many eukaryotic regulons

In our initial characterization of the membership of the RRB regulon in *S. cerevisiae* we noted that a highly significant fraction of the genes occurred in the genome as adjacent gene pairs [26]. This report extends that finding significantly, and reveals that this phenomena is not constrained to gene sets associated with ribosome biogenesis, but rather that a wide variety of other responsive gene sets in *S. cerevisiae* also contain significant numbers of adjacent gene pairs. While considerable attention has been paid to the identification and characterization of groups of genes that function in particular areas of metabolism, including the genes involved in the response to stress and nutrients [3], carbohydrate metabolism [33], nitrogen metabolism [34,35], toxic metals such as arsenic [36], the response to DNA damage [37], and the genes of the RP regulon [3], until now, the extent to which they include a significant fraction of adjacent gene pairing has been underappreciated. This non-random distribution of gene locations can be observed in even the smallest of gene sets, including the 8 member purine metabolism (62% adjacent), or response to arsenic (38% adjacent) gene sets, as well as in the 175 member DNA damage response (9% adjacent) set. When we did observe gene adjacency, it occurred as pairs of genes that were distributed across all possible orientations: divergent, tandem and convergent. There were cases in which up to three genes from within a given gene set were located in a row, but these were rare (roughly 3% of the genes), and there was only a single incidence of a four gene string (*IMA1-MAL13-MAL11-MAL12*). A recent report on the ‘neighboring gene effect’ provides additional evidence supporting transcriptional coupling of adjacent gene pairs on a genomic scale. A systematic screen of the yeast knock-out collection revealed individual gene deletions altered the regulated expression of the neighboring gene in about 10% of the cases [20].

The observation that considerable adjacent gene pairing can be recognized across a wide range of gene sets in divergent yeast species speaks to its evolutionary significance. Indeed there is a very high level of adjacent gene pairing in the RRB and RP regulons in distantly related yeast species (including as many as 24% of the RRB genes in *C. albicans*). Interestingly, while we can recognize distinct gene pairs in *S. cerevisiae* that have maintained their adjacency across many yeast species, and that the most closely related species tend to have a higher level of conservation of specific pairs, the more distantly related species have similar or greater overall levels of adjacent gene pairing, even if the exact pairs differ. Recently it has been reported that increased co-expression of neighboring gene pairs is retained even after their separation during evolution, and that newly formed gene pairs which arise from genomic rearrangements also tend to be co-expressed [38]. These findings were true for divergent, tandem and convergent gene orientations, and one possibility is that local chromatin remodeling processes act on gene pairs in a way that is distinct from unpaired genes. Our analysis indicates that the phenomena of adjacent gene coregulation preceded the whole genome duplication event, and is widely conserved across yeast species from *S. cerevisiae* to *S. pombe*, even though the exact pairing relationships - that is which gene is paired with which - are not. Indeed, by using the highly conserved and therefore easily recognized RP gene set as a test case, we found evidence for significant adjacent gene pairing across a wide range of eukaryotes, including in most of the well studied and well annotated systems. We propose that, like in yeast, other eukaryotes will also exhibit significant adjacent gene pairing in gene sets beyond those related to ribosome biogenesis.

### Paired genes exhibit a higher degree of transcriptional co-regulation than unpaired genes

Functionally, we observed that within a given set of related genes, those members that were present as immediately adjacent pairs exhibited a tighter degree of transcriptional co-regulation than the genes that were located on their own across the genome. Interestingly, this observation was true when expression profiles were compared between immediately adjacent genes, as well as when one gene of an adjacent pair was compared to another member of a distinct, adjacent gene pair. Thus, within a set of related genes, for example the 282 members of the RRB regulon in *S. cerevisiae*, the subset of the 44 paired genes are the most tightly co-regulated members of the regulon, even though the gene pairs themselves were scattered across the various chromosomes.

Interestingly, we also observed that the cases in which the specific pairing of adjacent genes was most widely conserved across divergent yeast species corresponded to those genes that were the most highly expressed [32]. Thus, there may be evolutionary pressure to favor adjacent gene pairing and concomitant transcriptional co-regulation for highly expressed and highly regulated genes. There may also be a connection between the extent of conservation of particular genomic arrangements, and the relative advantages of specific gene co-regulation in different ecological niches. For example, the observation that the specific gene pairings associated with the heat shock response are absent in *C. albicans* could be related to its relatively stable temperature environment as a human pathogen.

### Potential mechanisms for adjacent gene co-regulation

While further analysis of the *cis* and *trans* factors that mediate adjacent gene co-regulation will be required to elucidate how it is achieved, at least three, non-exclusive mechanisms can be proposed: 1) localized chromatin modification, 2) local DNA sequence looping, 3) co-localization of the genes to a common nuclear compartment. In terms of localized chromatin modifications, there is a correlation between genome-wide histone H3K14 acetylation and histone H4 acetylation domains that overlap with transcriptionally co-expressed genes in *S. cerevisiae*[39]. In higher eukaryotes, the transcriptional activation of one gene can result in a localized chromatin ‘opening’ that ultimately creates a more transcriptionally permissive transcriptional state [40]. This state can be propagated across significant distances, and it can affect the transcription of genes within a shared neighborhood [12]. In terms of DNA looping, it has been observed that elements of the *HMR-E* locus can impart silencing onto an adjacent gene via a local looping of DNA sequences that brings the promoter of the adjacent gene into physical contact with the *HMR-E* silencing factors [41]. By using the same chromosome conformation capture (3C) technique, genome-wide DNA looping interactions have been detected between genes on the same and different chromosomes in yeast [5] and, interestingly, co-regulated genes within similar ontologies were found to be preferentially associated with each other [42]. Finally, it is possible that adjacent gene co-regulation may be mediated, in part, at the level of sub-nuclear compartmentalization. High resolution mapping of gene localizations in yeast revealed that transcriptionally active sets of genes, including those involved in ribosome biogenesis, occupied specific nuclear territories at the nucleolar periphery upon activation [43]. In higher eukaryotes, active genes have been found to associate with discrete ‘transcription factories’, which are the site of nascent RNA production and are enriched for RNA pol II and associated transcription factors [44]. Therefore, if one member of a gene pair became localized to an active sub-nuclear compartment, the adjacent gene could potentially fall under the same regulatory umbrella.

## Conclusion

It appears that one of the ways that eukaryotic cells regulate the expression of genes within distinct regulons, or related pathways, is by distributing them, in part, as pairs of adjacent genes across the genome. The phenomena of adjacent gene co-regulation is widespread across eukaryotes, evolutionarily conserved, and functionally significant for maintaining coordinated levels of gene expression.

## Methods

### Calculating the average pairwise Pearson’s correlation coefficient from transcription profiles

The transcriptional similarity between two genes was calculated using the metric previously described [45] to calculate the Pearson’s correlation coefficient (PCC) between two genes, *X* and *Y* across a series of *N* conditions:

$$S\left(X,Y\right)=\frac{1}{N}\underset{i=1,N}{\Sigma }\left(\frac{X_{i}-X_{\text{offset}}}{\phi x}\right)\left(\frac{Y_{i}-Y_{\text{offset}}}{\phi y}\right)$$

where:

$$\phi G=\sqrt{\underset{i=1,N}{\Sigma }\frac{{\left(G_{i}-G_{\text{offset}}\right)}^{2}}{N}}$$

*G*_*offset*_ was set to the expression levels prior to perturbation or to the average expressional state (the reference state) in each dataset. Microarray datasets were downloaded from the Gene Expression Omnibus and transcription was monitored across two independent heat shock time-courses [3,28] (GEO accession numbers: GDS112 and GDS281), an osmotic shock time course [3] (GDS20), a timecourse following exposure to menadione [3] (GDS108), and a time-course following release from alpha factor synchronization [29] (GEO accession number: GDS38). The PCC scores for the unpaired genes represent the average of every possible pairing partner for every possible unpaired gene within the set. The PCC for the paired gene subset represents that average PCC score between each gene and every other paired gene, excluding that gene’s immediate adjacent neighbor. P-values were determined by bootstrapping with replacement, by taking at least 10,000 random groupings of genes (the same size as the paired subset) and determining the average PCC score for that grouping. The *p*-value was calculated from this distribution.

### Sets of genes that were analyzed in this study

In order to determine the frequency of adjacent gene pairing within *S. cerevisiae* we selected a total of 28 sets of functionally related genes for analysis. These sets were defined previously by their gene ontology and downloaded from the *Saccharomyces* Genome Database (see Additional file 8: Table S2 and Additional file 5 for a complete list of accession numbers and the genes within each group). The rationale behind the sets of genes that were chosen was to pick a representative cross-section of those pathways that are involved in metabolism and responding to the environment (and, thus, in maintaining cellular homeostasis). The groupings were selected to represent a wide range of ontology sizes, from up to 282 genes in the RRB regulon to the 8 member purine biosynthesis pathway.

### Determining the conservation of gene pairing relationships within the diverse fungal lineages

The pairing relationships for the *Saccharomyces sensu strictu* species (*Saccharomyces paradoxus, Saccharomyces mikatae*, and *Saccharomyces bayanus)* were determined based on synteny [46]. The pairing relationships for *Candida glabrata*, *Kluyveromyces lactis, Kluyveromyces waltii* and *Saccharomyces kluyveri* were determined using the Yeast Gene Order Browser and are based on synteny [47]. The pairing relationships for *C. albicans*, *C. dubliniensis* and *S. pombe* were determined based on homology [48,49].

### Determining the conservation of the RRB and RP gene pairing relationships within *Saccharomyces sensu strictu* species

A bootstrap analysis was performed to determine the conservation of adjacent gene pairs throughout *Saccharomyces sensu strictu* species. Starting with all the pairs of adjacent genes (*N*-1, where *N* is equal to the number of genes within the genome) within the *S. cerevisiae* genome a set of *S* genes was chosen (where *S* was either size of 282 or of 180) and conservation of their genomic arraignment was determined by looking within the *S. paradoxus*, *S. mikatae*, or *S. bayanus* genomes [50]. This analysis was run 10,000 times (with replacement after selection) for each set of genes and the percentage of paired genes is plotted against the frequency of occurrence.

### Identifying RRB homologues and calculating the statistical significance of gene adjacency

Ribosomal proteins were defined as all genes whose products are considered structural components of the ribosome (including those that are cytosolic, chloroplastic, apicoplastic and mitochondrial). The rRNA and ribosome biogenesis regulon in *S. cerevisiae* was defined as described previously [26], consisting of 188 genes, and was expanded based on the gene ontology terms: ribosome biogenesis, rRNA processing, 90S pre-ribosome and small subunit (SSU) processome. Once the redundant genes were removed we had expanded the RRB family to a set of 282 genes (see Additional file 11).

The homologues were identified for the genes of the RRB regulon using the WU-BLAST algorithm to search for conservation of the protein coding sequences from the *S. cerevisiae*[20]. The total number of genes used in the calculations included all verified protein coding genes from *H. sapiens*[51], *D. melanogaster*[52], *C. elegans*[53], *A. thaliana*[54], *T. thermophila*[55], *P. falciparum*[56], *G. lamblia*[57], *N. crassa*[58], *A. nidulans*[59], and *N. gruberi*[60]. The genomic distributions of these gene sets were manually curated. There were several instances where an RRB gene that was identified by BLAST homology was adjacent to a gene with a characterized function in ribosome biogenesis (but it was not one of the RRB set homologs we initially identified), but we did not include these genes in our statistical analysis (a complete list of these genes is provided in Additional file 10).

### Calculating the statistical significance of gene adjacency

To determine what significance there was to the genomic arrangement that we found, we calculated the statistical significance of this arrangement by using the binomial probability. The chance probability that there would be *j* adjacent genes within a regulon of size *M* genes is:

$$1-\overset{j}{\underset{k=0}{\Sigma }}\left(\frac{M!}{k!\left(M-k\right)!}\right)\left(p^{k}{\left(1-p\right)}^{M-k}\right)$$

where:

$$p=\left(\frac{M}{N}\right)\left(2-\frac{M}{N}\right)$$

and *N* is the total number of genes present within each species. The functional *p*-values were then calculated in Mathematica.

## Competing interests

The authors declare that they have no competing interests.

## Authors’ contributions

JTA contributed to the study design, participated in the data collection, helped with the data analysis, and helped to write the manuscript. ARP contributed with the data analysis and participated in the data collection. JRA and SKG both participated in the data collection. MM conceived the study and wrote the manuscript. All authors read and approved the final manuscript.

## Supplementary Material

Additional file 1 **Figure S1. **The transcription profiles of the entire set of paired RRB genes throughout a heat shock (A-F). For clarity, a maximum for three sets of pairs are plotted per graph. Click here for file

Additional file 2 **Figure S2. **The transcription profiles of the entire set of paired RP genes throughout a heat shock (A-C). For clarity, a maximum for three sets of pairs (or two sets of triplets) are plotted per graph. Click here for file

Additional file 3 **Figure S3. **The transcription profiles of the entire set of paired rRNA and ribosome biosynthesis genes following release from alpha-factor synchronization (A-F). For clarity, a maximum for three sets of pairs are plotted per graph. Click here for file

Additional file 4 **Figure S4. **The transcription profiles of the entire set of paired ribosomal protein genes following release from alpha-factor synchronization (A-C). For clarity only a maximum for three sets of pairs (or two sets of triplets) are plotted per graph. Click here for file

Additional file 5 **Complete list of the *S. cerevisiae *gene members for the various GO term groupings.** Click here for file

Additional file 6 **Table S1. **No significant gene pair adjacencies were found within the following ontologies. Click here for file

Additional file 7 **Figure S5. **The transcription profiles of the heat shock genes throughout a heat-shock induction of the budding yeast stress response. The unpaired members of the regulon are show on top (A) and the two sets of pairs are show in (B).(B) Click here for file

Additional file 8 **Table S2. **The closest homologs (E-value < 10 ^-9^) to members of the paired gene sets are not their immediate, adjacent neighbors. Click here for file

Additional file 9 **Figure S6.** Adjacently paired RRB and RRP genes are conserved in Saccharomyces sensu strictu species. Paired sets of genes were randomly selected and their conservation was determined by comparing that pairs’ location in *S. paradoxus*, *S. bayanus* and *S. mikatae.* Every gene pairing relationship that is conserved in any of these species was counted and the histogram showing frequency plot is shown for the set of pairs the size of the RRB regulon (n = 282) and for the RP regulon (n = 180) above (A and B, respectively). The actual percentage of conserved RRB (†) genes and RP (‡) genes is highlighted. Click here for file

Additional file 10 List of adjacent members for the RP and RRB gene sets in divergent eukaryotes. Click here for file

Additional file 11 **List of expanded RRB gene set membership from  *S.cerevisiae.*** Click here for file
