# Supplementary figures and images for: The adjacent positioning of co-regulated gene pairs is widely conserved across eukaryotes
Source: BMC Genomics. 2012 Oct 10;13:546. doi: 10.1186/1471-2164-13-546 (PMC3500266; doi:10.1186/1471-2164-13-546)

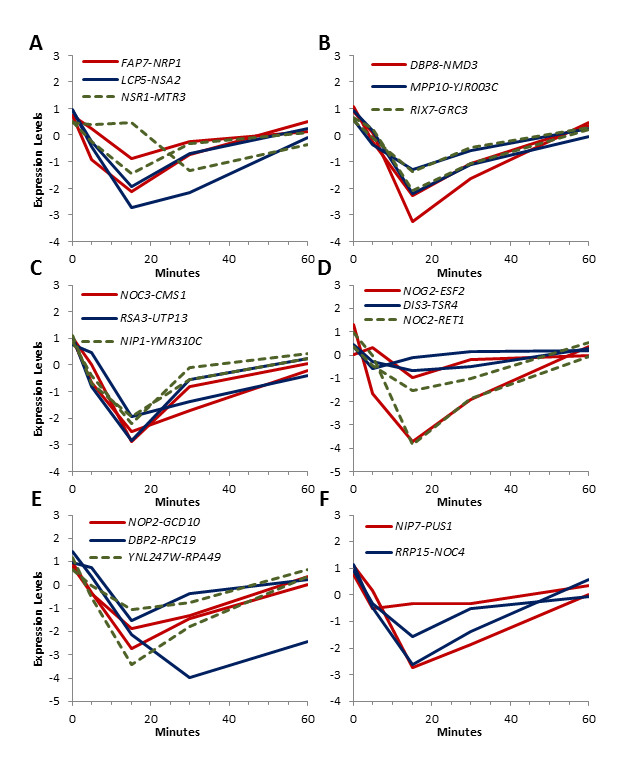

Supplement: Additional file 1 — Figure S1. The transcription profiles of the entire set of paired RRB genes throughout a heat shock (A-F). For clarity, a maximum for three sets of pairs are plotted per graph. [file 1471-2164-13-546-S1.tiff]

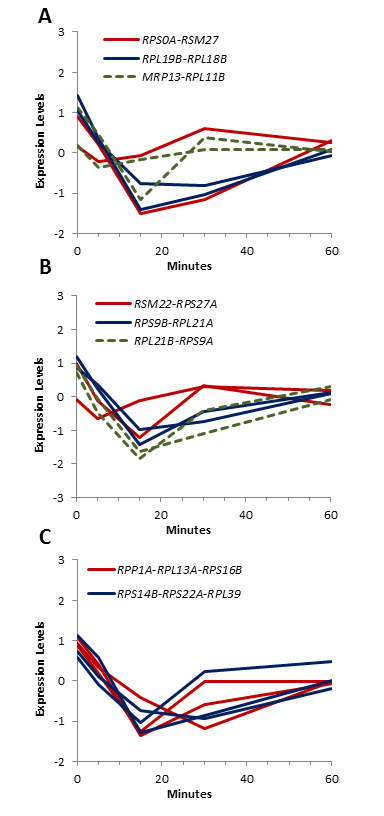

Supplement: Additional file 2 — Figure S2. The transcription profiles of the entire set of paired RP genes throughout a heat shock (A-C). For clarity, a maximum for three sets of pairs (or two sets of triplets) are plotted per graph. [file 1471-2164-13-546-S2.tiff]

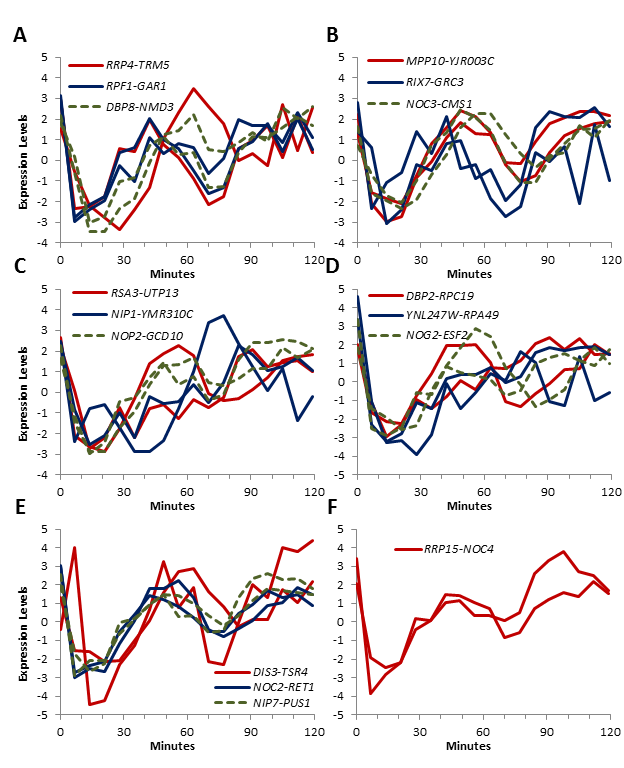

Supplement: Additional file 3 — Figure S3. The transcription profiles of the entire set of paired rRNA and ribosome biosynthesis genes following release from alpha-factor synchronization (A-F). For clarity, a maximum for three sets of pairs are plotted per graph. [file 1471-2164-13-546-S3.tiff]

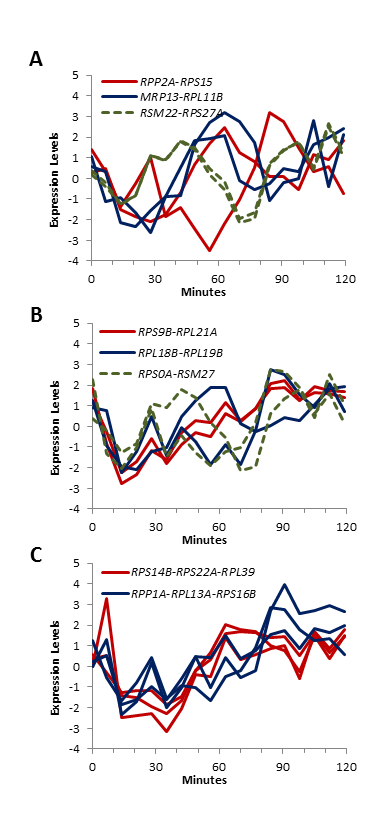

Supplement: Additional file 4 — Figure S4. The transcription profiles of the entire set of paired ribosomal protein genes following release from alpha-factor synchronization (A-C). For clarity only a maximum for three sets of pairs (or two sets of triplets) are plotted per graph. [file 1471-2164-13-546-S4.tiff]

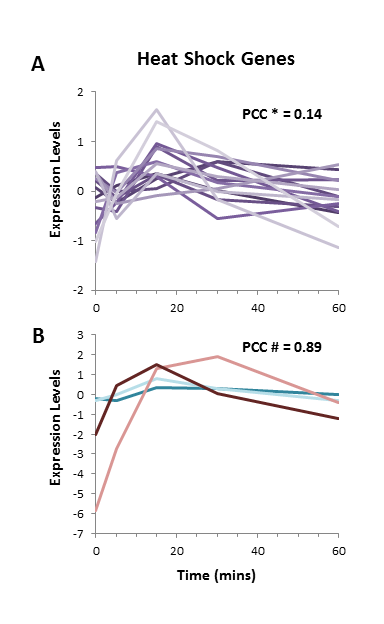

Supplement: Additional file 7 — Figure S5. The transcription profiles of the heat shock genes throughout a heat-shock induction of the budding yeast stress response. The unpaired members of the regulon are show on top (A) and the two sets of pairs are show in (B).(B) [file 1471-2164-13-546-S7.tiff]
