# Supplementary material for: The adjacent positioning of co-regulated gene pairs is widely conserved across eukaryotes
Source: BMC Genomics. 2012 Oct 10;13:546. doi: 10.1186/1471-2164-13-546 (PMC3500266; doi:10.1186/1471-2164-13-546)
Supplement: Additional file 6 — Table S1. No significant gene pair adjacencies were found within the following ontologies. [file 1471-2164-13-546-S6.doc]

Supplemental Table 1: No significant gene pair adjacencies were found within the following ontologies:

| **Ontology Class** | **G.O. Number** | **Number of Genes** |
| --- | --- | --- |
| Alcohol Metabolism | 0006066 | 46 |
| Cellular Metabolism | 0044237 | 15 |
| Cellular Respiration | 0045333 | 10 |
| Energy Reserve Metabolism | 0006112 | 28 |
| Phosphorus Metabolism | 0006793 | 47 |
| Sulfur Metabolism | 0006790 | 11 |
| Glycolysis | 0006096 | 31 |
| Osmotic Stress Response | 0006970 | 28 |
| Salt Stress Response | 0009651 | 15 |
| Oxidative Stress Response | 0034599 | 69 |
| Unfolded Protein Response | 0006986 | 11 |
| Protein Ubiquitinylation | 0016567 | 41 |
| Macroautophagy Response | 0016236 | 22 |
| Response to Acid | 0001101 | 9 |
| Drug Response | 0042493 | 24 |
| Pheromone Response | 0019236 | 37 |
| Water Response | 0009415 | 32 |
| Pyrimidine Nucleotide Biosynthetic Process | 0006221 | 10 |
