# Supplementary material for: The adjacent positioning of co-regulated gene pairs is widely conserved across eukaryotes
Source: BMC Genomics. 2012 Oct 10;13:546. doi: 10.1186/1471-2164-13-546 (PMC3500266; doi:10.1186/1471-2164-13-546)
Supplement: Additional file 8 — Table S2. The closest homologs (E-value < 10 -9) to members of the paired gene sets are not their immediate, adjacent neighbors. [file 1471-2164-13-546-S8.doc]

Supplemental Table 2: The closest homologs (E-value < 10 ^-9^) to members of the paired gene sets are not their immediate, adjacent neighbors.

| **Gene** | **Gene(s) with Significant Homology** | **E-value(s)** |
| --- | --- | --- |
| *GAL1* | *GAL3* | 1.2x10^-215^ |
| *IMA1* | *IMA2, IMA3, IMA4* | 0 |
| *ARO4* | *ARO3* | 3.1x10^-116^ |
| *SNO1* | *SNO2, SNO3* | 2.5x10^-75^, 5.2x10^-75^ |
| *SNZ1* | *SNZ2, SNZ3* | 1.4x10^-118^, 1.8x10^-118^ |
| *RPL30B* | *RPL40A* | 5.8x10^-67^ |
| *MLP1* | *MLP2* | 6.7x10^-100^ |
| *AAD4* | *AAD14, AAD10, AAD3,*  *AAD16, AAD6, AAD15* | 2.3x10^-168^, 1x10^-142^, 4.4x10^-140^, 4.2x10^-73^, 1x10^-62^, 3.5x10^-53^ |
| *THI13* | *THI5, THI11, THI12* | 5.6x10^-188^, 1.5x10^-187^, 1.5x10^-187^ |
| *RPL24A* | *RPL24B* | 1.2x10^-59^ |
| *RPL18A* | *RPL18B* | 2.3x10^-81^ |
| *RPL19A* | *RPL19B* | 2.6x10^-80^ |
| *RPP2A* | *RPP2B* | 2.3x10^-10^ |
| *RPS0A* | *RPS0B* | 7.1x10^-110^ |
| *RPL11B* | *RPL11A* | 6.6x10^-91^ |
| *RPS27A* | *RPS27B* | 4.7x10^-42^ |
| *RPS9B* | *RPS9A* | 3.6x10^-90^ |
| *RPL21A* | *RPL21B* | 8.4x10^-75^ |
| *RPS9A* | *RPS9B* | 3.6x10^-90^ |
| *RPP1A* | *RPP1B* | 1.1x10^-18^ |
| *RPL13A* | *RPL13B* | 2.2x10^-92^ |
| *RPS16B* | *RPS16A* | 1.1x10^-72^ |
| *RPS14B* | *RPS14A* | 9.4x10^-44^ |
| *RPS22A* | *RPS22B* | 9.5x10^-67^ |
